# Non-parametric multiple inputs prediction model for magnetic field dependent complex modulus of magnetorheological elastomer

**DOI:** 10.1038/s41598-022-06643-4

**Published:** 2022-02-17

**Authors:** Kasma Diana Saharuddin, Mohd Hatta Mohammed Ariff, Irfan Bahiuddin, Ubaidillah Ubaidillah, Saiful Amri Mazlan, Siti Aishah Abdul Aziz, Nurhazimah Nazmi, Abdul Yasser Abdul Fatah, Mohd Ibrahim Shapiai

**Affiliations:** 1 Malaysia Japan International Institute of Technology, Universiti Teknologi Malaysia, Jalan Sultan Yahya Petra, 54100 Kuala Lumpur, Malaysia; 2 Department of Mechanical Engineering, Vocational College, Universitas Gadjah Mada, Yogyakarta, Indonesia; 3 Mechanical Engineering Department, Faculty of Engineering, Universitas Sebelas Maret, Jl. Ir. Sutami 36A, Kentingan, Surakarta, 57126 Indonesia; 4 Institute for Vehicle Systems and Engineering (IVeSE), Universiti Teknologi Malaysia, Sultan Ibrahim Chan-Cellery Building, Jalan Iman, 81310 Skudai, Johor, Malaysia; 5 Razak Faculty of Technology and Informatics, Universiti Teknologi Malaysia, Jalan Sultan Yahya Petra, 54100 Kuala Lumpur, Malaysia

**Keywords:** Engineering, Materials science

## Abstract

This study introduces a novel platform to predict complex modulus variables as a function of the applied magnetic field and other imperative variables using machine learning. The complex modulus prediction of magnetorheological (MR) elastomers is a challenging process, attributable to the material’s highly nonlinear nature. This problem becomes apparent when considering various possible fabrication parameters. Furthermore, traditional parametric modeling methods are limited when applied to solve larger-scale cases involving large databases. Consequently, the application of non-parametric modeling such as machine learning has gained increasing attraction in recent years. Therefore, this work proposes a data-driven approach for predicting multiple input-dependent complex moduli using feedforward neural networks. Besides excitation frequency and magnetic flux density as operating conditions, the inputs consider compositions and curing conditions represented by magnetic particle weight percentage and the curing magnetic field, respectively. Extreme learning machines and artificial neural networks were used to train the models. The simulation results obtained at various curing conditions and other inputs confirm that the predicted complex modulus has high accuracy with an R^2^ of about 0.997, as compared to the experimental results. Furthermore, the predicted complex modulus pattern and magnetorheological effect agree with the experimental data using both the learned and unlearned data.

## Introduction

Magnetorheological (MR) elastomers are intelligent materials with tunable viscoelastic properties when exposed to magnetic fields^1^. MR elastomers are composed of matrix elements (e.g., silicone rubber and natural rubber)^2^ and commonly used micro-sized magnetic particles (e.g., carbonyl iron particles (CIPs))^3,4^. MR elastomers are suitable for vibration absorber applications due to their stiffness flexibility and damping properties in the presence of a magnetic field^5^. One example is the use of isolators in civil infrastructure as seismic protection to replace conventional isolators^6,7^. The MR effect of MR elastomers is an important consideration when designing a device because a higher MR effect means a more comprehensive controllability range^8,9^. When a sample is subjected to a range of magnetic field densities, the MR effect is calculated based on the measurement of the dynamic modulus. Many studies have proposed various methods to improve MR effect values, particularly the essential parameters, such as the variations of magnetic particle concentration^10,11^, curing magnetic field^12,13^, and magnetic particle shapes and sizes^14–16^. As discussed in recent works^17,18^, different curing conditions can affect material properties by changing the dispersion condition and distance between magnetic particles during the curing process. The curing condition classification can be anisotropic or isotropic with chain like-alignment in the presence of magnetic fields and arbitrary alignment in the absence of magnetic fields^19^.

When the effect of each fabrication process-related parameter is quite predictable, the pattern becomes more challenging if two or more parameters are considered^20^. For instance, Berasategi et al.^21^ demonstrated the effect of two parameters—CIP weight percentage and curing condition —on rheological properties and a highly nonlinear relationship between the variables. The anisotropic MR elastomers exhibit significant effects of up to 142%, whereas isotropic ones with 114% at a CIP weight percentage of 30%. Several methods, either in the microscopic approach or macroscopic approach, can be used to predict the behavior and pattern of the problem^13,22^.

Conventional viscoelastic models are one option for predicting the complex modulus. The viscoelastic behavior of MR elastomers is related to the strain–stress relationship on the given load, as represented by complex modulus measurement. The complex modulus is calculated from the sinusoidal waveforms of shear stress, ($$\tau )$$ and shear strain ($$\gamma )$$ over time with the specified phase shift (viscoelastic: $${0}^{^\circ }\le \delta \le {90}^{^\circ }$$). The ratio of stress to strain will be the complex shear modulus ($${G}^{*})$$^23^. Conventional viscoelastic models, such as the Kelvin Voight and Maxwell models, are commonly used to predict the properties of MR elastomers with minor structural modifications. These phenomenological models consist of a spring and a damper, which represent the stiffness and damping properties of MR elastomers, respectively. Three input variables are commonly mentioned to predict the complex modulus: strain amplitude, frequency, and magnetic flux density^24–28^. The previous viscoelastic model has limited inputs; therefore, it cannot be applied when other parameters must be considered. As an example, Tran et al.^29^ proposed a linear viscoelastic model, namely the fractional derivative model. Their samples were fabricated using a constant CIP volume of 27% at different curing conditions: isotropic and anisotropic. There are two particle distributions and hence, the data fitting for each isotropic and anisotropic sample must be assigned separately to obtain its parameter.

Even though conventional viscoelastic models are easy to handle, the accommodated inputs are limited. For example, Agirre et al.^30^ developed a new method to consider one more composition related-variable to predict the complex or dynamic modulus of MR elastomer. With the modification, the input variables include frequency, magnetic flux density, and matrix-filler concentration. A similar method is also applied to accommodate five CIP volume fractions^31^ for anisotropic conditions. However, developing a similar model to accommodate more input variables is time-consuming and requires careful consideration of the additional parameters. Therefore, a model will be beneficial if it can flexibly accommodate more inputs. Rather than conventional methods as part of the parametric models, non-parametric data or data-driven models are preferable solutions to consider more inputs, especially when dealing with a more extensive database.

Machine learning is one of the possible methods that has recently gained popularity in material prediction studies^32,33^. Previously, machine learning was applied to predict the viscosity and shear stress of MR fluids^34,35^, the tensile-compression mode in MR elastomer^36^, and MR device behavior^37–40^. Previous works also only described the relationship accommodating basic characterization input variables without considering the fabrication process^41,42^. To the best of the authors' knowledge, modeling approaches that simultaneously accommodate curing conditions, ingredients, and magnetic field density have never been reported in the literature.

Therefore, this work proposes a multi-input model using a non-parametric approach that uses machine learning as a potential solution to predict the complex modulus and magnetorheological effect at various curing conditions and compositions that can be considered as multiple outputs cases. The workflow is described as follows. First, the modeling structure of the feedforward neural network is formulated and describes the training algorithm, which is an artificial neural network (ANN) and an extreme learning machine (ELM). Then, the sample preparation and data collection are carried out. Finally, the simulation results are discussed through several analyses before summarizing all sections.

## Proposed model formulation

### Model development

The dynamic viscoelastic properties of MR elastomer can be affected by various inputs, such as strain amplitude, frequency, magnetic fields, and composition under isotropic and anisotropic conditions. Strain amplitude and frequency are the primary independent input variables for investigating the dynamic characteristics of MR elastomer. In addition, the magnetic field is a distinctive independent input variable for magnetic materials, such as MR elastomer. These three input variables can be remarked as the characterization input using a rheometer. The characterization process is carried in^43^ by using a ramped magnetic field with strain amplitude and excitation frequency conducted via oscillation test.

Fabrication conditions also affect the dynamic properties of MR elastomers. The fabrication conditions can be curing magnetic flux density value, magnetic field direction, and temperature. The applied magnetic field direction can influence the particle alignment lock during curing conditions. Consequently, the alignment has a significant effect on the dynamic modulus of MR elastomer^21^. Curing conditions are classified into two types: isotropic and anisotropic. Isotropic is a condition where the magnetic particle is randomly distributed in the matrix. In contrast, anisotropic is aligned in a chain by applying a certain magnetic field during curing conditions. Both conditions may produce different MR effects and dynamic modulus. In addition, different applications used different curing conditions following its usages. Thus, it is important to consider both isotropic and anisotropic conditions when analyzing the dynamic modulus of MR elastomer.

The composition is another variable that has been proven to affect the MR elastomer. The matrix-filler composition on MR elastomer would have a significant effect on the dynamic modulus. The addition of filler or magnetic particle dispersed in matrix rubber, such as CIP, improves dipole–dipole or interparticle magnetic forces and interfacial slipping among adjacent CIPs and rubber matrix as particle distances close^44^. In addition, the stiffness and damping properties of an MR elastomer are greatly dependent on the distance and interfacial slipping or friction between the matrix rubber and the magnetic particle^45,46^. As the CIP concentration increases, more energy is stored and dissipated within the MR elastomer sample^44^. However, different compositions are needed for other applications. Hence, it is crucial to clarify that the composition or concentration of magnetic particles is important to determine the viscoelastic properties of MR elastomer. Aside from these two fabrication conditions, variables such as magnetic particle shapes or sizes have an impact on the rheological properties of MR elastomer. However, those variables are currently constant. Yet, the machine learning model is a data-driven approach that has been carried out to train with massive amounts of data. There are two ways to improve the generosity of a machine learning model. New data can be combined with the existing data, and the training process will be conducted in similar ways. A new model can also be developed with the same function or training algorithm. This avoids the need for the complex development of a new formula for a new problem using a parametric approach.

The three groups of variables represent the factors that influenced the dynamic modulus of MR elastomer to be accommodated in the formulated model. The characterization process is represented by strain amplitude, frequency, and magnetic field in predicting dynamic states on oscillation tests. This work also provides insight on the effect of excitation frequency on-ramp magnetic field specifically to resolve the vibration issue. A strain amplitude of 0.01% is chosen due to the linear viscoelastic (LVE) region in which the microstructure is considered linear due to strong bonding between particle and matrix^47^. Hence, the variables used to represent the characterization processes are frequency $(f)$ and magnetic flux density $(B)$. Besides, the particle distribution, which is isotropic or anisotropic, is also included as an independent variable in the model, represented by the value of the applied magnetic field during curing conditions $({B}_{c})$. Then, the concentration of magnetic particle $({W}_{p})$ representing the composition is also accommodated.

**Figure 1:**
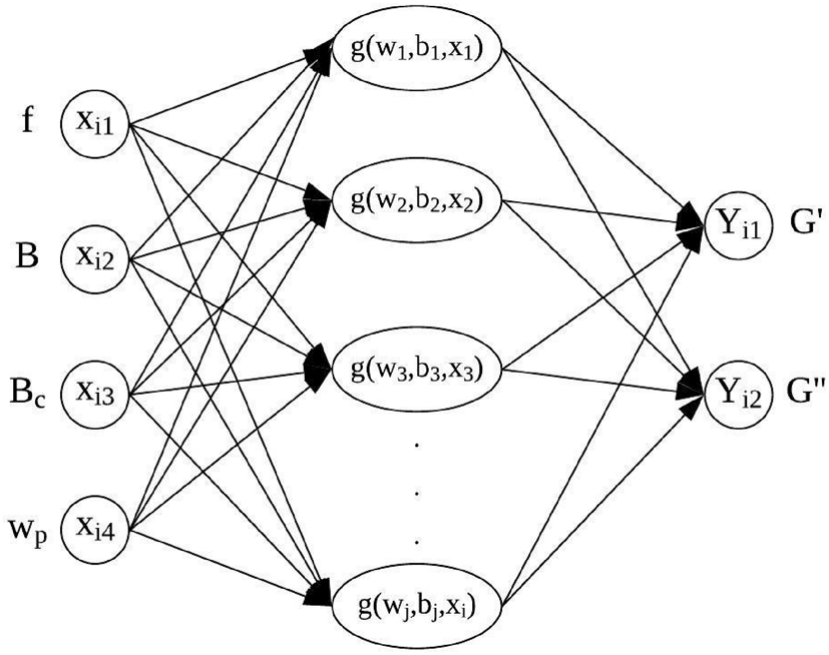
The SLFN structure for MR elastomer viscoelastic properties.

A neural network model can be formulated by considering the aforementioned variables with four inputs and two outputs. The inputs are $f$, $B$, ${B}_{c}$, and ${W}_{p}$. The outputs are the storage modulus ($G{^{\prime}}$) and loss modulus ($G{^{\prime\prime}}$). The chosen neural networks structure consists of a single hidden layer feedforward neural network (SLFN). Figure 1 depicts the general SLFN structure.

### Machine learning methods for predicting MR elastomer viscoelastic properties

The proposed model can be trained using various machine learning methods or learning algorithms, such as ELM and ANN. ANN may have many variations in network architecture, but in this work, the multilayer perceptron (MLP) architecture is being used due to its easy implementation, understanding, and low complexity^36^. Meanwhile, there are a few steps to describe the ELM structure, which was introduced by Guang Bin Huang^48^ and is known for its fast prediction and accuracy comparable with ANN^49^.

## Back propagation artificial neural networks (BP-ANN)

The backpropagation (BP) learning algorithm is used in MLP architecture as a supervised learning method. During the supervised learning method, the output network is compared with the known desired output^50^ to determine how well the network is working. The BP-ANN training is carried out in MATLAB using the neural network toolbox. The Levenberg Marquardt (LM) training algorithm is used as a training algorithm because most applications practically on viscoelastic material^51–53^ agreed that it could produce better results while also requiring less training time. The BP-ANN algorithm repeatedly adjusted the weight and bias of network parameters to reduce the cost function between predicted output and experiment output. The LM training algorithm minimizes the cost function by using Guess Newton combined with the gradient descent method to update the weight and bias of the network.

1 $${\mathbf{w}}_{k+1}={\mathbf{w}}_{k}-[{\mathbf{J}}^{T}\mathbf{J}+{\mu \mathbf{I}]}^{-1}{\mathbf{J}}^{T}\mathbf{e}$$

where ${\mathbf{w}}_{k+1},$ ${\mathbf{w}}_{k},$ J, e, $\mu ,$ and $\mathbf{I}$ represent the updated weight, current weight, Jacobian matrix, vector error, a scalar, and identity matrix, respectively.

## Extreme learning machine (ELM)

The ELM main steps are as follows:

1. Randomly assigned input weight, ${w}_{i}$ and bias, ${b}_{i}$ were $i=\mathrm{1,2},3,\dots ,\stackrel{\sim }{ N.}$

2. Calculate the hidden layer output matrix, $\mathbf{H}.$

3. Calculate the output weight, $\beta$*.*

In the first step, the Gaussian distribution or normal distribution can be applied as distribution to define the weight and bias. Then, for the second step, the value of hidden node output at the $j\mathrm{th}$ hidden node number and $i$ th sample (${h}_{j, i}(f,{ B, W}_{p},{ B}_{c})$ for all hidden nodes or to define the hidden layer output matrix, $\mathbf{H}$ as expressed in Eq. (2).

2 $$\mathbf{H}={\left[\begin{array}{ccc}g({w}_{1},{b}_{1},{f}_{1},{B}_{1},{W}_{p,1},{B}_{c,1})& \cdots & g({w}_{L},{b}_{L},{f}_{1},{B}_{1},{W}_{p,1},{B}_{c,1})\\ \vdots & \ddots & \vdots \\ g({w}_{1},{b}_{1},{f}_{N},{B}_{N},{W}_{p,N},{B}_{c,1})& \cdots & g({w}_{L},{b}_{L},{f}_{N},{B}_{N},{W}_{p,N},{B}_{c,N})\end{array}\right]}_{N\times L}$$

where N is the number of samples, whereas for the third step, the formula in calculating the output weight, $\beta$, is expressed in Eq. (3).

3 $${\varvec{\beta}}={\mathbf{H}}^{\dagger}\mathbf{T}; \mathbf{T}=\left[\begin{array}{c}{T}_{1}\\ \cdots \\ {T}_{N}\end{array}\right],{\varvec{\beta}}=\left[\begin{array}{c}{\beta }_{1}\\ \cdots \\ {\beta }_{L}\end{array}\right]$$

where T is the storage modulus and loss modulus of the experimental value or target for the training process. ${\mathbf{H}}^{\dagger}$ is the Moore–Penrose generalized inverse (pseudoinverse)^54^ of matrix $\mathbf{H}$**.**

In a machine learning model, training data is required to develop the model. Furthermore, because supervised learning is used, ANN and ELM should know the respected inputs and outputs to be used for training purposes. The input variable consists of the factors that affect the MR elastomer performances, as mentioned in the previous section, which are frequency, magnetic field, curing condition, and magnetic field compositions. Meanwhile, the outputs for this work are storage modulus and loss modulus, which are used to represent the viscoelastic properties of MR elastomers, notably on dynamic modulus. Moreover, the model hyperparameters are hidden nodes and activation functions. These two parameters will be varying to have the optimum model based on the input–output relationship. The input–output relationship is represented by the general function, as shown in Eq. (4). The storage modulus and loss modulus are the outputs of the model represented by $G_{i} ^{\prime}$ and $G_{i} ^{\prime\prime}$, respectively.

4 $$G_{i} ^{\prime}\,,G_{i} ^{\prime\prime}=f\left({f}_{i},{B}_{i},{B}_{{c}_{i}},{W}_{{p}_{i}}\right)$$

SLFN has three layers: an input layer, a hidden layer, and an output layer. In this network, the input variables, ${f}_{i},{B}_{i},{B}_{{c}_{i}},{W}_{{p}_{i}},$ and the output variables $G_{i} ^{\prime}\,,G_{i} ^{\prime\prime}$ will be assigned in the input and output layers, respectively. Meanwhile, in the hidden layer, all possible hidden neurons and activation functions will be assigned, as discussed in the next section. There are several steps involved to train the model. Both ANN and ELM have similar processes except for the learning structure. The process starts with 1. Initialize the input and output data; 2. Distribute the data into training and testing; 3. Assign model hyperparameters, such as hidden nodes and activation function. The activation function of hidden neurons varies frequently, like that of the output layer. However, most of the applications assign the Purelin function in the output layer. 4. Train the data based on the assigned model hyperparameter. 5. Validate the developed model using unlearned data. 6. Evaluate the model’s performance by calculating the error between experimental data and simulation data.

## Materials

### Material fabrication

The silicon rubber and carbonyl iron powder were used as the main components of MR elastomer. The room temperature vulcanization silicon rubber (RTV-SR) with a density of 1.08 g/cm^3^was purchased from Nippon Steel (NS625) supplied by Nippon Steel Co., Japan. The tensile strength for RTV-SR is $\ge 4.4 MPa,$ with $\ge 450$% of elongation at break. The hardness and viscosity are 25 $\text{\AA}$ and 18 Pa$\pm 2$-s at 25 °C, respectively. Meanwhile, carbonyl iron powder, which consist of 99.5% iron (Fe), 0.05% carbon (C) and 0.01% nitrogen (N), 0.18–0.35% oxygen (O) with spherical shape utilized as a magnetic particle with size 3.8–5.3 $\mu m$, CC grade, was purchased by BASF, Germany. Furthermore, the curing agent, NS625B (Nippon Steel), was used as a cross-linking agent ($\approx 0.1 \mathrm{wt}\%).$

The CIP’s composition has been varied with five weight percentages, 30%, 40%, 50%, 60%, and 70%. However, several works showed that the materials with CIP concentrations less than 30 wt% had a narrow range of shear modulus over magnetic field variations and low self-assembled microstructure^44,55^. In addition, the MRE sample with 30 wt% shows its stability after thermal aging^56^. Furthermore, it is found in^2,11^ that most of the previous works used 70 wt% as its maximum concentration. The mixtures were vigorously stirred for about ten minutes to ensure homogeneity. Following that, the compounds were cured using a cylindrical mold with a thickness of 1 mm. This work has included isotropic and anisotropic particle distribution. For isotropic distribution, the mold was kept at room temperature for about two hours, whereas for anisotropic distribution, the mold was kept in the presence of a magnetic field fixed at 300 mT for all of the anisotropic MR elastomer samples. Table 1 details the composition for all prepared MR elastomer samples.

**Table 1:** The prepared MR elastomer samples.

Sample	Silicon rubber (%)	CIP (wt%)	Curing condition (magnetic field strength; mT)
ISO-S1	70	30	0
ISO-S2	60	40	0
ISO-S3	50	50	0
ISO-S4	40	60	0
ISO-S5	30	70	0
ANISO-S6	70	30	300
ANISO-S7	60	40	300
ANISO-S8	50	50	300
ANISO-S9	40	60	300
ANISO-S10	30	70	300

### Oscillation test

The dynamic rheological properties of fabricated MR elastomer were measured using a rheometer (MCR 302 Anton Paar, Germany). The MRD 170/1 T magneto cell is equipped with the rheometer to generate a magnetic field so that the testing can be done in the presence of a magnetic field. When the current flows through the coil, the magnetic field will be guided go through the sample. A tesla meter is used to measure the magnetic field. Table 2 provides the current equivalent to the magnetic flux density. Before testing, the samples were cut using a 20 mm-diameter cylindrical film to fit the rotating disk parallel plate rheometer. Thus, the tested sample has a diameter of 20 mm and a thickness of 1 mm. Three dynamic tests were done to observe the characterization of rheological properties of MR elastomers.

**Table 2:** The current applied equivalent to magnetic flux density.

Current (A)	Magnetic flux density (T)
0	0
1	0.18
2	0.37
3	0.58
4	0.69
5	0.81

#### Strain amplitude sweep testing

In this testing, the shear strain amplitude was set from 0.001 to 25% for about 30 points. Meanwhile, the frequency was set at 1 Hz. The magnetic field strength was fixed on six values, which are on the off-state condition (0 T) and on-state condition (0.18, 0.37, 0.58, 0.69, and 0.81 T).

#### Current sweep testing

In this testing, the applied magnetic field strength was varied from 0 to 0.9 T for 30 points. The frequency was set to 1 Hz, while the shear strain amplitude was set to 0.01% due to its linear viscoelastic region found on strain amplitude sweep testing.

#### Frequency sweep testing

In this testing, the frequency was varied from 0.1 Hz to 10 Hz for 30 points. The shear strain was set to 0.01%, while the magnetic field strength was fixed on six values for on–off state (0 T) and on-state (0.18, 0.37, 0.58, 0.69, and 0.81 T).

## Data sets and simulation setups

The total sample, N, in this work consists of 2160 data points with 70 data sets in total. For the information related to data points, the minimum requirement for the data number for training can be different for each case. For instance, in nanofluid materials, Adio et al.^57^ used only 154 data points whereas Gholami et al.^58^ used 540 data points. On the other hand, the method’s application in magnetorheological elastomer can be considered rare. Meanwhile, there is no restrain on how many data points should be used to train the model; it depends on the network performance and data set distribution. The data distribution process should be carried out carefully for achieving a good network performance and avoiding issues such as underfitting and overfitting. In the modeling approach, the data must be divided into three parts: training, validation, and generalization. The validation technique used in this work is the hold-out method for both ANN and ELM models in which can eliminate biasness of data and reduced training time. The modeling training phase includes the training and validation parts. These two parts are known as modeling data. Meanwhile, the generalization part is used to determine the generalization performance of the model. For modeling data, the data for the training and validation part is randomly distributed to 80% and 20%. Twenty percent of modeling data is being used as validation data sets to check the performance of the model, which is near to the input of training data^59^. In contrast, the generalization part is far or outside of training data that can be spotted as “unlearned” data. As for the generalization part, there are four data sets: Ts2, Ts3, Ts4, and Ts5, which represent unlearned magnetic flux density data, unlearned CIPs composition data, unlearned MR effect data, and a combination of all unlearned data, respectively.

This section describes the simulation setup for ANN and ELM. The training algorithm used in ANN is LM with one hidden layer. Then, the number of the hidden node varies from 2 to 10. The tangent sigmoid is used as an activation function in the hidden layer, whereas pure linear is used in the output layer. As for the ELM modeling method, the hidden layer is made up of one layer where the weight and bias are updated randomly. Furthermore, the activation function used in hidden nodes varies into three types: the Sigmoid function, Sine function, and Hard limit function. As for hidden nodes number, the typical values are 10, 100, 1000, 10,000, and 55,000. The training process of the model is repeated about three times for each activation function and hidden node number to gain consistency. Table 3 compiles these parameters.

**Table 3:** Network tuning parameter.

Network parameters	ANN	ELM
Training algorithm	Levenberg Marquardt	Extreme learning machine
Hidden layer number	1	1
Hidden nodes number	2,3,4,5,6,7,8,9,10	10,100,1000,10,000, 55,000
Weight and bias determination	Levenberg Marquardt	Randomly distribute
Activation function	Tangent sigmoid	Sigmoid, Sign, Hard limit

The accuracy for these models is measured through the performance index, which is the root mean square error (RMSE) and coefficient of determination (R^2^). Equations (5) and (6) show the calculation of RMSE and R^2^, respectively.

5 $$RMSE= \sqrt{\frac{1}{n}\sum_{i=1}^{n}{({Y}_{i}-{\widehat{Y}}_{i})}^{2}}$$

6 $${R}^{2}=1-\frac{{\sum }_{i=1}^{n}{({Y}_{i}-{Y}_{i})}^{2}}{{\sum }_{i=1}^{n}{({Y}_{i}-{\widehat{Y}}_{output})}^{2}}$$

**Figure 2:**
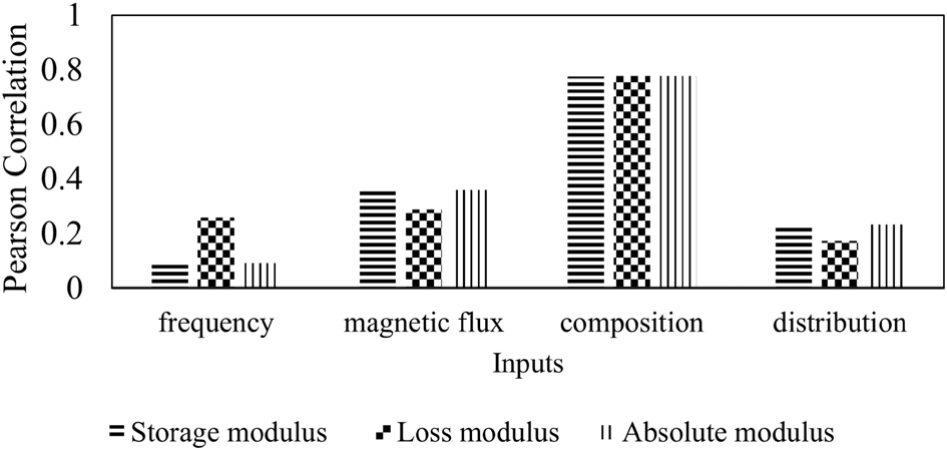
Correlation analysis between input and output variables.

After determining the input and output variables, the correlation between variables must be identified. In this work, the Pearson correlation analysis is used to investigate the correlation between the input to input and between the input to output. The best input variable may have a higher correlation to the output, hence having a big impact on the performance of the model. Furthermore, two inputs that have a high correlation with each other indicate that the two inputs are redundant. Meanwhile, if two inputs have zero correlation, the input variables are independent of each other. Based on Table 4, almost all input variables have low correlation. That means there is no redundancy between the two inputs for this work. Figure 2 presents the correlation between input and output variables. There are three output variables used to investigate the correlation analysis: storage modulus, loss modulus, and absolute modulus. However, the absolute modulus is not considered as model output because it can be derived from the storage and loss modulus values. Based on the figure, the composition ${(W}_{{p}_{i}}$) has a higher correlation for all outputs for about 0.77 when compared to other inputs. Note that the highest value of correlation is + 1, and the lowest is − 1. The second higher is magnetic flux density, for about 0.36 for storage and absolute modulus, and 0.29 for loss modulus. The second-lowest is particle distribution input variables. Notice that the loss modulus has the lowest correlation when compared to storage and absolute modulus. Unexpectedly, loss modulus has a high correlation with the frequency input, whereas storage and absolute modulus have the lowest correlation for about 0.1. In general, all input variables are correlated with outputs even though there exists a low correlation coefficient such as between frequency and storage modulus or particle distribution and loss modulus. A low Pearson correlation coefficient does not imply that the variables cannot be considered in the modeling process or that there is no relationship^60^. The variables may have a nonlinear relationship where will be investigated further in the future. Thus, all input variables are considered in this work to model the viscoelastic behavior.

**Table 4:** The correlation between input variables.

Inputs	Frequency	Magnetic flux density	Composition	Particle distribution
Frequency	1	− 0.1112	1.44E−14	− 1.32E−14
Magnetic flux density	− 0.1112	1	0.016	0.0042
Composition	1.44E−14	0.016	1	− 1.917E−14
Distribution	− 1.32E−14	0.0042	− 1.917E−14	1

## Results and discussion

### Prediction of complex modulus

**Figure 3:**
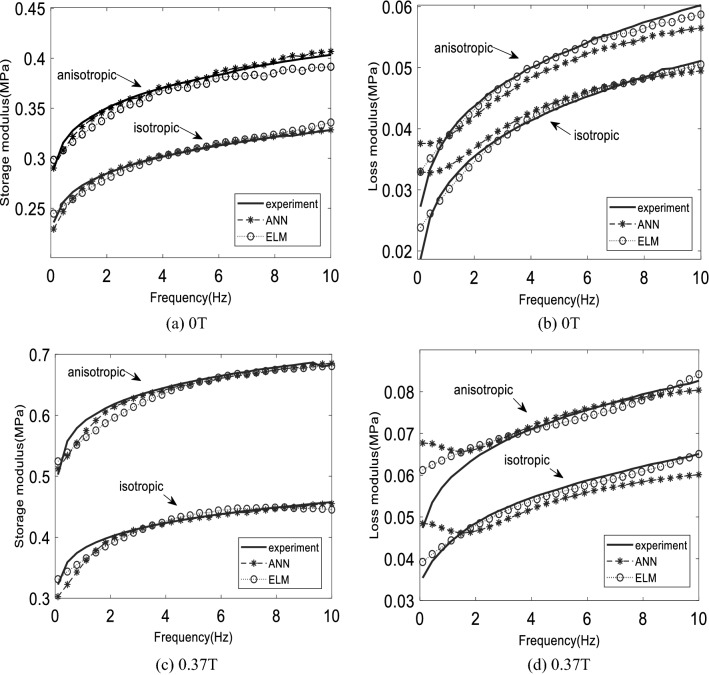
The comparison of dynamic moduli between experimental, ANN, and ELM model on 70% CIP where (**a**), (**b**) for learned 0 T, (**c**), (**d**) for learned 0.37 T for both distributions.

**Figure 4:**
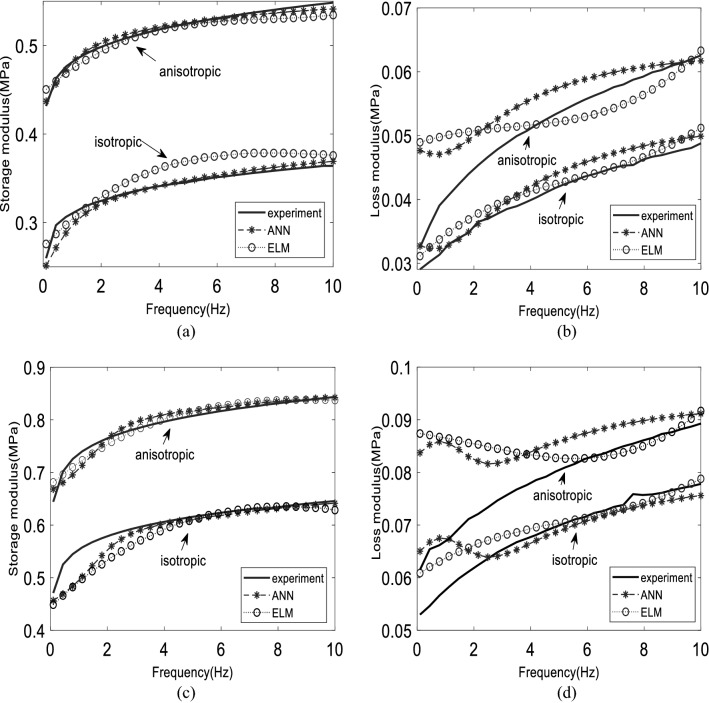
The comparison of dynamic moduli between experimental, ANN, and ELM model on 0.69 T: (**a**), (**b**) for learned 60% CIP, (**c**), (**d**) for learned 70% CIP on both distributions.

**Figure 5:**
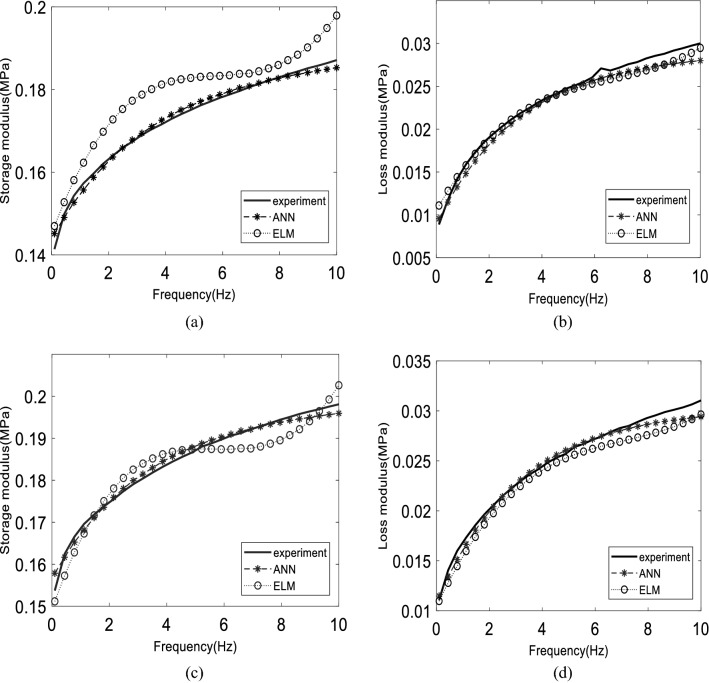
The comparison of dynamic modulus between experimental, ANN, and ELM model for Ts2 data at 0.58 T, 30% where (**a**), (**b**) for isotropic distribution and (**c**), (**d**) for anisotropic distribution.

**Figure 6:**
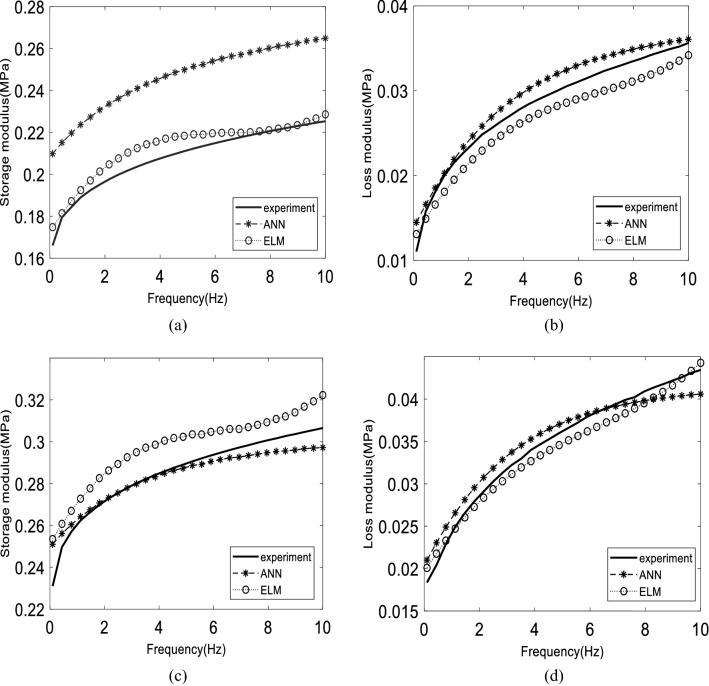
The comparison of dynamic moduli between experiment, ELM, and ANN model for Ts3 data set at 0.37 T, 50% of CIP: (**a**), (**b**) for isotropic distribution and (**c**), (**d**) for anisotropic distribution.

This section discusses the performance of the proposed model based on listed data in Table 5. The effect of excitation frequency on rheological properties of MR elastomer, specifically complex shear modulus, is being predicted by two proposed models based on a machine learning approach, by comparing with experimental data. Table 6 shows the accuracy of two configurations of ANN models and four configurations of ELM models that have potential in terms of accuracy compared to other configurations based on hidden nodes number and activation function. ANN-8 and ANN-10 have the highest accuracy among other ANN configurations, where ANN-8 and ANN-10 denoted 8 and 10 hidden nodes, respectively. On the other hand, ELM-SINE1 and ELM-SINE2 denoted 10,000 and 55,000 hidden nodes with sine activation function whereas ELM-SIG1 and ELM-SIG2 represented 10,000 and 55,000 hidden nodes with a sigmoid activation function, respectively. Table 6 displays the accuracy for all data. According to the RMSE and R^2^ values, the ELM-SINE2 model has higher accuracy for almost all data sets than other ELM models, whereas, for the ANN model, the ANN-8 model may have better generalization performance than the ANN-10 model. In addition, Ts5 data sets that represent the combination of all unlearned data have the highest accuracy among all models. This proved that the ELM-SINE2 model is the most generous among other models. Figures 3, 4, 5 and 6 depict the results of these models.

**Table 5:** Data division for isotropic and anisotropic MR elastomer.

Part	Data sets	Data points	Magnetic flux density (T)	CIPs composition (wt%)	Remarks
Training	Tr	960	0, 0.18, 0.37, 0.69	30, 40, 60, 70	80% of modeling data
Validation	Ts1	240	0, 0.18, 0.37, 0.69	30, 40, 60, 70	20% of modeling data
Generalization	Ts2	600	0.58, 0.81	30, 40, 50, 60, 70	Unlearned magnetic flux density
	Ts3	360	0, 0.18, 0.37, 0.58, 0.69, 0.81	50	Unlearned CIPs composition
	Ts4	60	Range (0–0.9)	50	Current sweep
	Ts5	1020	0, 0.18, 0.37, 0.58, 0.69, 0.81	30,40,50,60,70	Combination of unlearned data

**Table 6:** The accuracy of various configurations for the proposed models.

Data	ELM-SINE1		ELM-SINE2		ELM-SIG1		ELM-SIG2		ANN-8		ANN-10	
	RMSE	R^2^	RMSE	R^2^	RMSE	R^2^	RMSE	R^2^	RMSE	R^2^	RMSE	R^2^
Tr	0.0091	0.997	0.0072	0.998	0.0137	0.994	0.0131	0.994	0.0053	0.999	0.0041	0.999
Ts1	6.59e-5	0.998	5.018e-5	0.998	1.64e-4	0.995	1.95e-4	0.993	0.0057	0.998	0.0055	0.999
Ts2	0.0133	0.996	0.0109	0.997	0.210	0.990	0.0199	0.991	0.0118	0.996	0.0182	0.992
Ts3	0.0069	0.997	0.0064	0.997	0.0108	0.995	0.0097	0.996	0.0171	0.979	0.0224	0.971
Ts4	0.0069	0.997	0.0079	0.997	0.0073	0.997	0.0074	0.996	0.0185	0.973	0.0233	0.967
Ts5	0.0111	0.996	0.0095	0.997	0.0174	0.991	0.0165	0.992	0.0143	0.994	0.0201	0.988

Significant values are in bold.

The implementation of MR elastomer can be seen in various applications such as vibration absorbers^61^ and acoustic devices^62^, which involve frequency variation to utilize the functionality of MR elastomer in dynamic properties. Thus, before it is enforced in these devices, it is crucial to investigate its rheological properties by using a frequency sweep test. This section discusses the modeling performance of complex modulus, specifically on storage and loss modulus based on excitation frequency from 0.1 to 10 Hz. Generally, the complex modulus linearly increases with the applied frequency for both isotropic and anisotropic MR elastomer. Meantime, the stiffness of the MR elastomer increases as the CIP compositions and the magnetic field strength are increased.

As shown in Table 7, the best ANN and ELM models, which are ANN-8 and ELM-SINE2, were used to compare the performance on the separated dynamic moduli, storage modulus, and loss modulus. The RMSE value for data sets that include training, and a series of testing data is more specific compared to Table 6. In general, the ANN model produced a low RMSE value on the training data set, especially on storage modulus. Meanwhile, the RMSE value for loss modulus is not too significant, with a difference between the ANN and ELM models being only 0.001 MPa. Figure 3 shows the model’s performance for the training data set. Figure 3a,b show the storage and loss modulus on 0 T (off-state), respectively. Meanwhile, Fig. 3c,d present both storages as well as loss modulus for 0.37 T, respectively. From the figure, the anisotropic distribution has produced a larger storage modulus compared to the isotropic distribution. The larger storage modulus is due to the anisotropic MR elastomer possessing a closed gap between magnetic particles compared to isotropic. Thus, it is much more sensitive towards the changes in magnetic field intensity^63^. In addition, it does not require much energy to form the aligned structure of the magnetic field direction. Hence, the energy observed by storage modulus is higher compared to isotropic distribution that needs more energy to be aligned. The prediction of the ANN and ELM models on storage modulus is stable, for 0 and 0.37 T. Yet, the prediction of ANN on loss modulus has a small error, practically at low frequency, 0 to 2 Hz. This might be due to a nonlinear phase in which the models were struggling to follow the pattern. This is obvious for isotropic distribution, where the error is much higher compared to anisotropic. However, this does not mean that the neural network is unable to forecast nonlinear cases. This needs more thorough studies on related tuning hyperparameters such as activation function and learning rare as one of the solutions to reduce the error at the particular response. Figure 4 depicts a more graphical evaluation of the training data set. Figure 4 presents the storage and loss modulus for 0.69 T of magnetic flux density. A slight error occurred on ELM prediction exhibited in Fig. 4a for isotropic distribution. This may have contributed to the high RMSE value stated in Table 7. Furthermore, increasing the magnetic field increases the error prediction, based on loss modulus, mainly in anisotropic distribution. It happens on higher CIP composition. Even though the error seems to be larger on visual observation, the RMSE value for the loss modulus is still lower than the storage modulus. This is due to the magnitude storage modulus being one time larger than the loss modulus^31,64^. Thus, the analysis should be carefully done when dealing with a small magnitude.

**Table 7:** The RMSE value for each data set.

Data	ANN-8		ELM-SINE2	
	Storage modulus (MPa)	Loss modulus (MPa)	Storage modulus (MPa)	Loss modulus (MPa)
Training (Tr)	0.006	0.003	0.009	0.002
Testing 2 (Ts2)	0.016	0.004	0.015	0.005
Testing 3 (Ts3)	0.024	0.178	0.009	0.002
Testing 4 (Ts4)	0.026	0.004	0.011	0.004
Testing 5 (Ts5)	0.020	0.004	0.013	0.004

The accuracy for Ts2 data sets is almost similar on both models. As shown in Fig. 5, which represents a part of Ts2 data, the ANN model presents excellent consistency compared to the ELM model for both storage and loss moduli. The ELM model produced a wavy pattern on isotropic (Fig. 5a) and anisotropic distribution (Fig. 5c). To summarize the accuracy for each Ts2 data set, Table 8 shows the RMSE value of absolute complex modulus for Ts2 data set on each unlearned magnetic flux density on five CIP weight percentages. In Table 8, the RMSE values of ANN (8hn) and ELM (55000-SINE) models were placed beside each other so that they could be easily compared. From the table, there is a pattern where the error increased by the increment of CIP weight percentages, especially when it reached 70 wt%. where it occurred on both models, at isotropic and anisotropic distributions. Furthermore, the RMSE also increased from interpolation data (0.58 T) to extrapolation data (0.81 T). This is obvious for the isotropic case. Meanwhile, no trend can be described in the anisotropic case. Interpolation data is data placed between the training data ranges, whereas extrapolation data is data located outside of the training data ranges. In general, ANN mostly has higher accuracy except for 50 wt% for isotropic for both 0.58 T and 0.81 T in which ELM can maintain lower accuracy for these data.

**Table 8:** The RMSE of absolute complex modulus on Ts2 data sets.

CIP weight percentage	30 wt.%		40 wt.%		50 wt.%		60 wt.%		70 wt.%	
Magnetic flux density	ANN	ELM	ANN	ELM	ANN	ELM	ANN	ELM	ANN	ELM
**Isotropic**										
0.58 T	0.0012	0.0068	0.0024	0.0040	0.0241	0.0044	0.0051	0.0073	0.0136	0.0195
0.81 T	0.0058	0.0068	0.0024	0.0150	0.0344	0.0058	0.0133	0.0371	0.0239	0.0291
**Anisotropic**										
0.58 T	0.0013	0.0033	0.0035	0.0100	0.0084	0.0166	0.0066	0.0075	0.0191	0.0112
0.81 T	0.0037	0.0100	0.0063	0.0051	0.0040	0.0079	0.0124	0.0115	0.0405	0.0197

On the other hand, the RMSE value for the Ts3 data set of the ELM prediction model, mentioned in Table 7, is lower than the ANN model, generally for both moduli. Figure 6 depicts the results, where ELM shows better performance practically for storage modulus and isotropic distribution (Fig. 6a).

In contrast, the ANN model produces better than the ELM model for anisotropic distribution (Fig. 6c). For the loss modulus, both ELM and ANN models have similar performances. These results indicate that by increasing the magnetic field, the model error becomes more significant. Moreover, the prediction on anisotropic distribution is erratic, starting at low frequency. This had a significant effect on loss modulus. To be more specific, Table 9 presents the RMSE values for each magnetic flux density on unlearned 50 wt% for Ts3 data sets. From the table, the ELM model outperforms the ANN model in which the RMSE values decreased by increasing the magnetic flux density until it stopped at 0.81 T. Moreover, no pattern can be observed for ANN models. On the other hand, the ANN model produced better performances for anisotropic distribution compared to the ELM model practically starting at 0.18 T. In addition, trends were indicating that the RMSE values increased until 0.58 T, then it decreased to 0.81 T. This occurred on both models. To summarize, no models can represent the best one for Ts3 data sets in which the ELM model is great at predicting the isotropic case and the ANN model performed better at the anisotropic case. A detailed analysis is explained in the next section to investigate more about the effect of input variables.

**Table 9:** The RMSE of absolute complex modulus on Ts3 data sets.

Particle distribution	Isotropic		Anisotropic	
	50 wt.%			
Magnetic flux density	ANN	ELM	ANN	ELM
0 T	0.0348	0.0128	0.0130	0.0046
0.18 T	0.0390	0.0063	0.0026	0.0030
0.37 T	0.0383	0.0054	0.0058	0.0124
0.58 T	0.0241	0.0044	0.0084	0.0166
0.69 T	0.0257	0.0042	0.0071	0.0096
0.81 T	0.0344	0.0058	0.0040	0.0079

#### Boxplot analysis

A boxplot analysis is carried out to study the effectiveness of the input variable. We analyzed the effects of three input variables—magnetic flux density $(B)$, CIP composition $({W}_{p})$, and particle distribution ${(B}_{c})$. The standard error of absolute modulus ($G^{*} = G^{\prime} + iG^{\prime\prime}$) was used to compare the capability of the ANN and ELM models using a boxplot. The boxplot is used to show the distribution of data for a continuous variable. In this work, the larger the boxplot represents the wider the error distribution for a model.

**Figure 7:**
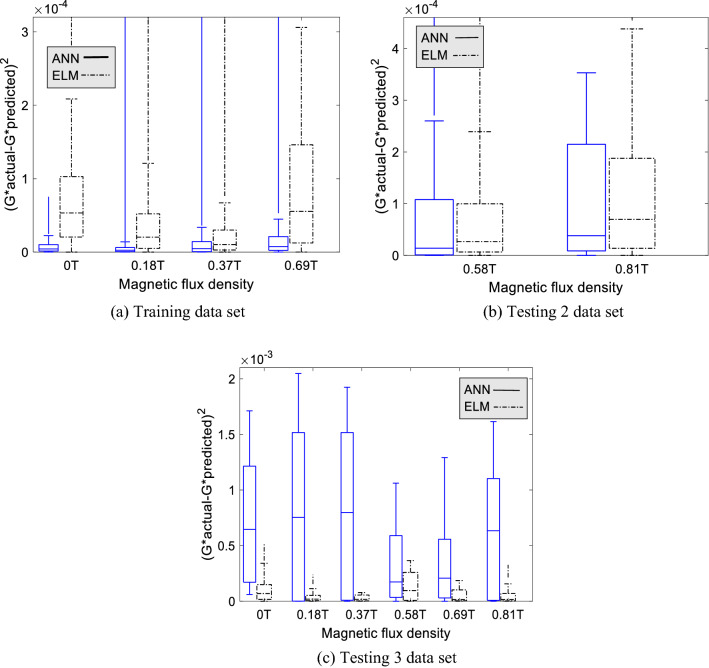
The boxplot of standard error for ANN and ELM model for three data set at particular magnetic flux density.

The boxplot for magnetic flux density sensitivity is shown in Fig. 7a–c. Generally, the ANN model has produced small error distribution for all training magnetic flux density, compared to ELM. The smallest error is at 0.18 T, where the error distribution approaches zero. Meanwhile, the median line of the ELM model boxplot is mostly higher than the maximum error of ANN, showing that the error distribution for ELM is wider than the counterparts. However, the ELM boxplot deteriorates as increasing the magnetic flux density, until it suddenly increases at 0.69 T. Furthermore, the ANN model also has a wider error distribution at 0.69 T compared to other training magnetic flux densities. This is due to the presence of a data gap between 0.37 and 0.69 T. Hence, the model may mispredict the data pattern because of dynamic modulus for 0.69 T is two times higher than 0.37 T. Meanwhile, the boxplot on Ts2 data is broadened its size as it increased the magnetic flux density. In this case, both models exhibit similar performances for both interpolation (0.58 T) and extrapolation data (0.81 T). Furthermore, the error on the Ts3 data set is higher than the Ts2 data set. In addition, there is a trend at Ts3 data for the ELM model where the boxplot size is decreasing starting from 0 until 0.37 T, but it increases again at 0.58 T and decreases again until 0.81 T. This also decided that the ELM model performs better at lower magnetic flux densities. The unexpected higher error distribution occurs on ANN for Ts3 data at almost all magnetic flux densities. In general, ANN showed overfitting phenomena in predicting the dynamic modulus on the effect of magnetic flux densities. The error distribution for the ELM model is controllable, notably on testing data.

**Figure 8:**
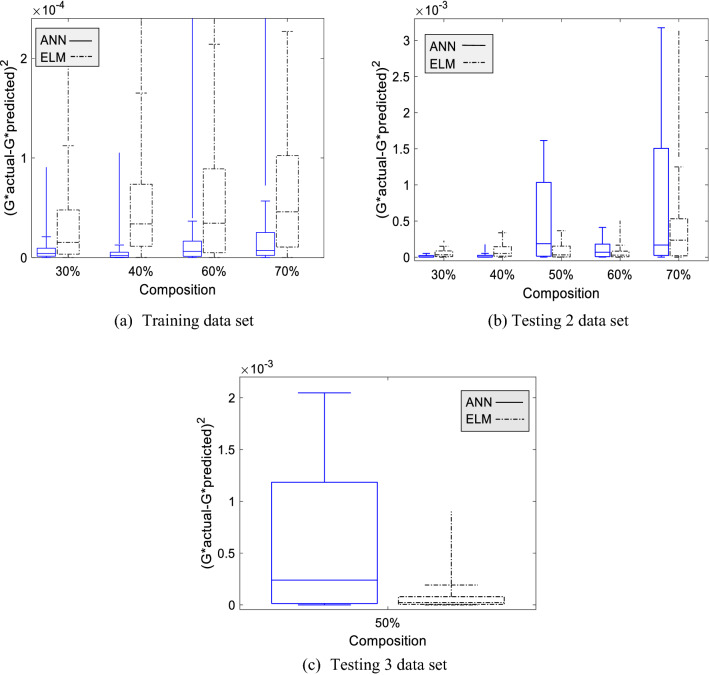
The boxplot of standard error for ANN and ELM model for three data set at particular CIP composition.

The boxplot on CIP composition sensitivity is explained in Fig. 8a–c. Similar to the previous case, the ANN model preceded the accuracy for the training data set compared to the ELM model for all trained compositions. Meanwhile, the ELM boxplot became wider as the compositions increased. Even though the error distribution of the ELM model becomes wider as increasing the composition, the transition of boxplot size between 40% CIP and 60% CIP is not too significant, though there is a data gap. The boxplot size for ANN at 60% CIP is two times larger than 40% CIP, indicating that the ANN model is complicated by data changes. The boxplot at Ts2 data set for ANN model, particularly at 30% CIP and 40% CIP is very small, described that the error for all data points is almost the same. Meanwhile, the ELM model produced a small error distribution even though it was not excellent as the ANN model error distribution. The ANN model exhibits wider error distribution at 50% CIP at Ts2 and Ts3 data set. Meanwhile, the error distribution for the ELM model at 50% CIP keeps maintaining a small range. The boxplot at 70% CIP shows the highest error distribution for both models. Nevertheless, the ANN boxplot is wider than the ELM boxplot and has a higher maximum error. Overall, the error distribution of the ELM model has a lower range compared to the ANN model as increasing the CIP composition, notably for testing data.

**Figure 9:**
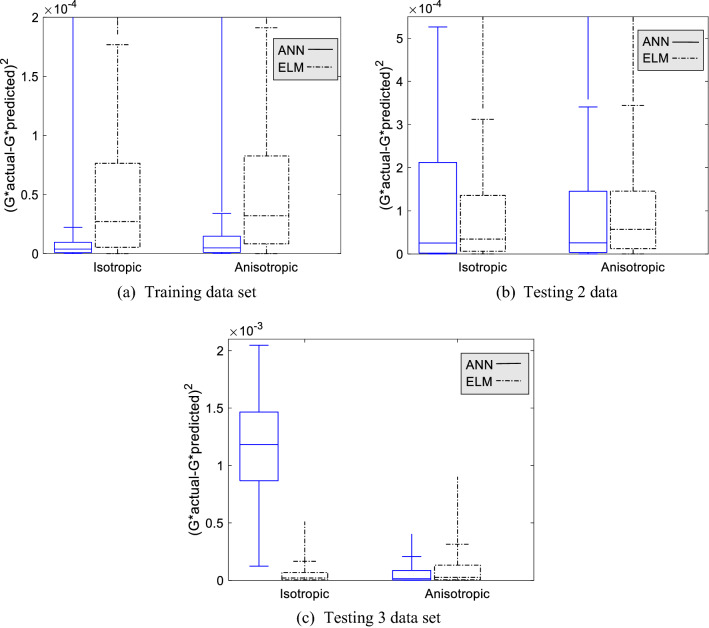
The boxplot of standard error for ANN and ELM model for three data set at the isotropic and anisotropic distributions.

The boxplot showing the error distribution for isotropic and anisotropic sensitivity is presented in Fig. 9a–c. The ANN model monopolizes the small boxplot compared to the ELM model for training data set for both particle distributions. Notice that the error distribution of the ELM model between isotropic and anisotropic is almost similar. It happens on the Tr and Ts2 data sets. Meanwhile, the error distribution for isotropic for the ANN model is more prominent than anisotropic. It was evident in the Ts3 data set, where the error range is more significant than anisotropic. Also, the placement of the median line in the middle explained that the distribution is normal. For the Ts2 data set, the error distribution is the same for both models, notably on anisotropic. Yet, ANN has a lower median line than the ELM model, indicating that most of the error in the ANN boxplot is small. In general, the ANN model exhibits good performance for anisotropic, for all data sets. Nevertheless, the ELM model is not deviating too much between isotropic and anisotropic compared to the ANN model.

Generally, the effect of three input variables on dynamic moduli, magnetic flux density, composition, and particle distribution on three data sets is identified. Mainly, as higher the magnetic flux density and composition, the error becomes apparent. Furthermore, the isotropic distribution exhibits a more significant error compared to anisotropic. The ANN model exhibit outstanding performance in predicting training data set for all input variables compared to the ELM model. However, the results on Ts2 and Ts3 data set are contrary to the training data. It has occurred for all input variables. Yet, ANN performed better at Ts2 compared to Ts3 data sets.

Meanwhile, the ELM model did not exhibit the overfitting phenomena for all input variables. To conclude, the boxplot for the majority of input variables is skewed to the right with a more extended part above the median. When the boxplot is right-skewed, it is explained that the error is small. However, the discussion must be fully covered by including the outlier’s data points. Yet, it should be done on the next project. This analysis is sufficient to show that the ELM model is considered a potential viscoelastic model to predict the dynamic modulus as a function of frequency, magnetic flux density, composition, and particle distribution. However, the training process should be improved to get better generalization performances.

**Figure 10:**
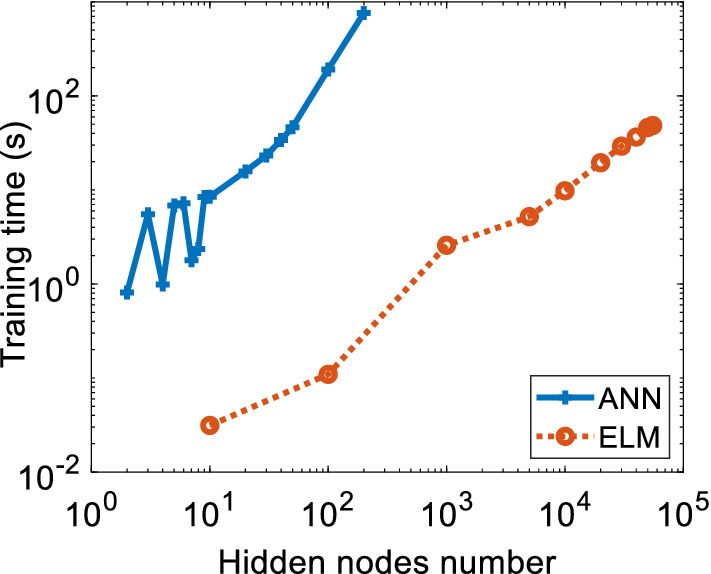
The training time for applied hidden nodes of ANN and ELM model.

Furthermore, training time analysis is crucial in determining the practical model. An overly longer training time is impractical, but flash training time may bring the overfitting issue, which lowers the accuracy of the machine learning model. In the SLFN network model, the hidden node numbers significantly impact the model performances. The higher the number of the remote node, the longer the training time of models. Figure 10 presents the training time versus hidden nodes number for ANN and ELM models. For the ANN model, the hidden nodes start with two until 200. Meanwhile, for the ELM model, the hidden nodes start from 10 until 55,000 hidden nodes. Figure 10 demonstrates that ANN took a longer training time, which is 764 s with 200 hidden nodes, while the ELM with 55,000 hidden nodes only took 48 s to execute.

**Figure 11:**
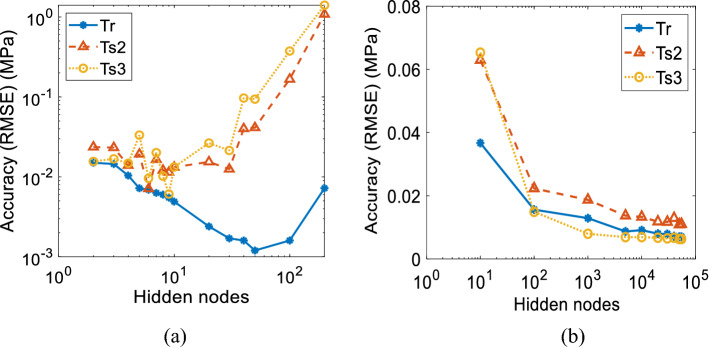
The training and testing accuracy for applied hidden nodes of (**a**) ANN model and (**b**) ELM model.

The accuracy for training and testing data is examined for the applied hidden nodes. The accuracy trend in terms of RMSE on the ANN and ELM model is plotted in Fig. 11, where Fig. 11a depicts the ANN model and Fig. 11b illustrates the ELM model. In Fig. 11a, the training error decreases as the number of hidden nodes increases. However, the testing accuracy shows the opposite trend for all testing data that starts on nine hidden nodes, where the error keeps increasing as the number of hidden nodes increases. Another significant finding is that the ANN model exhibits high accuracy within 6 to 9 hidden nodes, whereas above that, the model's performance is insignificant. These results indicate that the ANN model faces an overfitting issue when increasing the number of the hidden node.

Meanwhile, the higher the accuracy, the number of the hidden node for the ELM model must follow. It happens on training and testing data. In the future, there is no emphasis on increasing the number of the hidden node because the accuracy keeps maintaining notably on 50,000 hidden nodes. To conclude, the ELM model performs better in predicting MR elastomer's dynamic modulus than the ANN model based on its performances in accuracy and training time. Meanwhile, the ANN model is capable of predicting learned data better than the ELM model. Thus, the ANN model would be used as a prediction system for learned data to maintain its advantages.

### Magnetorheological (MR) effect

The observation of the MR effect can be done by the current sweep test by using the storage modulus. The relative MR effect was calculated using Eq. (7) by setting up a strain amplitude of 0.01% and a frequency of 1 Hz. The MR effect shows the changes in rheological properties of MR elastomer in the response of magnetic field strength^65^. MR effect can be influenced by many factors, such as the type of matrix rubber^63^, particle's shape^14^, size^10^, and volume content of particles^66^. Furthermore, studies have shown that by increasing the composition of CIPs in MR elastomer formulation, the interparticle interaction between magnetic particles becomes more assertive as more CIPs loaded due to an increase in magnetic force among CIPs^67^.

7 $$Relative\,MR\,effect= \frac{{G}_{max}-{G}_{0}}{{G}_{0}}\times 100\%$$

In addition, it is stated that anisotropic distribution has produced a more significant MR effect than isotropic distribution^21^. It is because anisotropic MR elastomer possessed a closed gap between magnetic particles compared to isotropic. Thus, it is much more sensitive towards the changes in magnetic field intensity^63^. Therefore, predicting the MR effect is crucial in determining the sample’s sensitivity towards magnetic field strength. Table 10 shows the value of relative MR effect for actual value, prediction from ANN and ELM models. The comparison is made for isotropic and anisotropic distribution. It is noted that the training CIP composition value is 30%, 40%, 60%, and 70% for both particle distributions. Meanwhile, 50% composition is the Ts4 data set used for generalization purposes.

**Table 10:** The comparison of relative MR effect between experiment and proposed models.

Wt.%	Isotropic			Anisotropic		
	EXP	ANN	ELM	EXP	ANN	ELM
30	17.6	16.8	22.1	28.9	37.6	25.8
40	30.0	30.9	14.6	34.3	41.2	33.6
50	47.2	**1.34**	28.8	62.3	66.9	66.0
60	62.1	59.3	69	94.9	121.7	100.4
70	105.7	105	115.2	126.8	138	141.1

**Figure 12:**
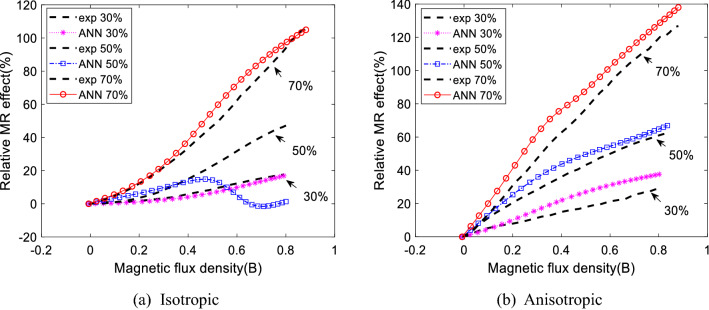
The comparison of relative MR effect between experimental and ANN model.

The ANN model produced an almost literal value of the relative MR effect on isotropic distribution for all training compositions from Table 10. Unfortunately, the prediction relative MR effect on testing data with a 50% CIP deviates too much. From Fig. 12a, the relative MR effect diverges from 0.4 T until the end. Meanwhile, the relative MR effect produced by the ELM model on isotropic distribution is not too impressive. Meanwhile, prediction on anisotropic distribution is not so favorable for both models. Figure 12b shows that the ANN predicts the actual MR effect on anisotropic distribution. Despite its low accuracy compared to isotropic, the simulation results can follow the pattern without overlapping with other CIP compositions. Notice that ELM is more satisfying on several CIP compositions: 30%, 40%, 50%, and 60% on predicting the relative MR effect. However, the calculation from the RMSE tabulated in Table 11 contradicts results precisely on 40% CIP and 50% CIP. It produced a closed relative MR effect to the actual value, yet the RMSE shows low accuracy. It is due to irregular patterns and results in which the model only produced an accurate result at maximum and minimum moduli. At the same time, the pattern deviates in the middle. In general, it can be concluded that the ANN model performed better on predicting the MR effect based on current sweep data than the ELM model. However, it is encouraged for the user to use the data trained to ensure the error is low.

**Table 11:** The RMSE of relative MR effect.

Wt.%	Isotropic		Anisotropic	
	${RMSE}_{ANN}$	${RMSE}_{ELM}$	${RMSE}_{ANN}$	${RMSE}_{ELM}$
30	1.55	3.51	7.33	2.23
40	0.51	8.00	4.64	10.61
50	24.65	9.89	4.98	11.36
60	1.48	4.47	23.54	4.17
70	5.60	7.58	10.43	15.38

## Conclusion

MR elastomer was fabricated in two conditions, isotropic and anisotropic on five CIP compositions. The viscoelastic properties were investigated, practically on complex modulus under different loading variables—strain amplitude, frequency, and magnetic field. As a result, the complex modulus of MR elastomer increases as CIP compositions increase. In addition, anisotropic exhibits a higher modulus than isotropic distribution, especially on higher composition. The BP-ANN and ELM machine learning models were developed to emulate the complex modulus on excitation frequency function. The model predicts the storage and loss modulus on excitation frequency under the influence of magnetic fields for different CIP concentrations in both isotropic and anisotropic conditions. Various configurations were developed during the training phase by using different activation functions and hidden nodes number. The performances of BP-ANN and ELM models were compared to experimental results based on RMSE and R^2^. As the result, ANN with 8 hidden nodes and ELM with sine activation with 55,000 hidden nodes produced high accuracy among other configurations for each method. Based on boxplot analysis, the BP-ANN model is excellently predicted on Tr data. Yet, BP-ANN had an overfitting issue where most of the prediction accuracy was poor, based on boxplot analysis of each input variable for Ts2 and Ts3 data sets. Yet, Ts2 accuracy was better than Ts3. It can be proved from the RMSE value where BP-ANN produced 0.003 MPa at Tr data set, while 0.178 MPa for Ts3 data set. Meanwhile, the ELM model maintained the performance where the RMSE values for Tr and Ts3 data sets are 0.002 MPa and 0.002 MPa, respectively. But, a bit higher on the Ts2 data set. Moreover, the capability of the proposed model is shown in the MR effect. In this condition, the prediction should be accurate. It is sensitive to the changing of maximum and minimum modulus properties. Thus, distraction will occur in determining the right compositions. In this case, BP-ANN is a good model due to its high performance in predicting the relative MR effect. Yet, the user should consider the compositions that have been trained. Overall, different working conditions in predicting the viscoelastic behavior of MR elastomer require different training algorithms. Nevertheless, more working condition is needed to determine specifically which method is appropriate. Meanwhile, many improvements can be made to the current method to enhance the accuracy of the model, especially on standardizing and normalizing the data. The storage and loss moduli have different significant values. Therefore, the model outputs also need to be standardized and normalized. Furthermore, in terms of dynamic performance, input such as curing magnetic flux density on anisotropic conditions and temperature can be added as the input model. Such inclusion will not only make the prediction model more inclusive, but it will also be beneficial for a wider range of applications.

## Data Availability

The data that support the findings of this study are available from the corresponding author upon reasonable request.
